# Rotate Vector (RV) Reducer Fault Detection and Diagnosis System: Towards Component Level Prognostics and Health Management (PHM)

**DOI:** 10.3390/s20236845

**Published:** 2020-11-30

**Authors:** Ali Rohan, Izaz Raouf, Heung Soo Kim

**Affiliations:** Department of Mechanical, Robotics and Energy Engineering, Dongguk University-Seoul, 30 Pil-dong 1 Gil, Jung-gu, Seoul 04620, Korea; Alirohan@dongguk.edu (A.R.); izazraouf@dongguk.edu (I.R.)

**Keywords:** prognostics and health management (PHM), fault detection and diagnosis, feature selection, machine learning

## Abstract

In prognostics and health management (PHM), the majority of fault detection and diagnosis is performed by adopting segregated methodology, where electrical faults are detected using motor current signature analysis (MCSA), while mechanical faults are detected using vibration, acoustic emission, or ferrography analysis. This leads to more complicated methods for overall fault detection and diagnosis. Additionally, the involvement of several types of data makes system management difficult, thus increasing computational cost in real-time. Aiming to resolve that, this work proposes the use of the embedded electrical current signals of the control unit (MCSA) as an approach to detect and diagnose mechanical faults. The proposed fault detection and diagnosis method use the discrete wavelet transform (DWT) to analyze the electric motor current signals in the time-frequency domain. The technique decomposes current signals into wavelets, and extracts distinguishing features to perform machine learning (ML) based classification. To achieve an acceptable level of classification accuracy for ML-based classifiers, this work extends to presenting a methodology to extract, select, and infuse several types of features from the decomposed wavelets of the original current signals, based on wavelet characteristics and statistical analysis. The mechanical faults under study are related to the rotate vector (RV) reducer mechanically coupled to electric motors of the industrial robot Hyundai Robot YS080 developed by Hyundai Robotics Co. The proposed approach was implemented in real-time and showed satisfying results in fault detection and diagnosis for the RV reducer, with a classification accuracy of 96.7%.

## 1. Introduction

**Figure 1:**
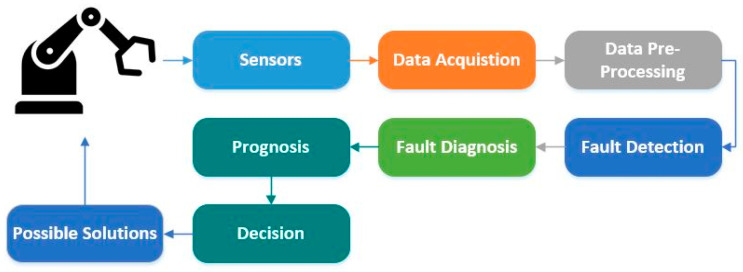
Basic architecture of a PHM system.

With the advancement in the field of automation and control, modern industrial systems have adopted programmed robotics arrangements to execute tasks autonomously with minimum human interference. These robots act as basic building blocks in the automation of industrial systems, and over time, their continuous operation in manufacturing processes causes the degradation of constituent sub-systems and components. Without proper maintenance, this degradation can create several faults in the system, which in turn causes unexpected shutdowns and production loss to the manufacturers. To address this, researchers are creating novel health monitoring, diagnostics, prognostic, and maintenance strategies that are collectively known as prognostics and health management (PHM). PHM is a mechanism of preventive measures that deliver comprehensive and tailored solutions for the industrial health system, management, and prediction. In industrial systems, health refers to a certain industrial application or component’s condition, efficiency, and remaining useful lifetime (RUL). PHM can be seen as a systematic approach to effective and productive health management systems [1]. PHM combines the detection of an initiating fault (fault detection), isolation, recognition of its origin, fault type (fault diagnostics), and the prediction of the remaining useful life (prognostics). Figure 1 shows the basic architecture of the PHM system. PHM has become a critical method to identify system failures that can cause major damage to the environment and the user. It has emerged as an integral means of delivering a competitive advantage in the international market by enhancing efficiency, sustainability, security, and accessibility. In recent years, ideas and components of the PHM under different names have been developed independently in various fields that include mechanical, electrical engineering, and statistical science. 

PHM can be utilized at both the component and system levels. Generally, the component level PHM focuses on monitoring the health of individual components (such as electrical and electronic devices, mechanical reducers, and engines), taking into account environmental, operational, and performance-related parameters to determine if the health of the monitored component is degraded by the time [2,3]. Meanwhile, the system level PHM analyzes the overall system health by taking into account the system architecture, system function, and process-related parameters [4]. Over the past years, there has been sustained research activity in PHM, especially with the immense development in the field of artificial intelligence (AI), to where it is now possible to create methodologies that utilize the decision-making capabilities of AI tools, such as deep learning (DL) and machine learning (ML), to develop a fault diagnostics and prognostics system in an efficacious way. Several researchers have been working on the design of such a system at both component and system levels. PHM methods can be categorized generally as either mathematical model-based or data-driven [5,6,7]. The mathematical model-based methods include knowledge of the basic principles of the object under inspection, such as material properties and structural features [8,9], whereas data-driven methods extract the information from the empirical data, to predict the health of a certain component or system [10,11]. With the easy availability of industrial data of different components and systems, data-driven methods, including DL and ML, are gaining popularity among PHM applications.

In particular, DL is considered to be well equipped in providing solutions for issues such as large-scale data processing [12] and the automatic derivation of meaningful features [13], as well as transferring knowledge between operating conditions and results. As many researchers have applied DL to PHM applications, they have significantly focused on fault diagnosis or prognosis [14,15], while others focus on applications to a specific item, such as a bearing or electronic system [16,17,18]. However, when it comes to commercialization and real-time implementation of DL methods, computational cost becomes much higher than typical ML methods, because of the huge volume of data involved in the extensive feature extraction and learning process. In a recent study published by MIT(Massachusetts Institute of Technology) [19], the computational limits of DL were analyzed, resulting in a claim that the computational requirements of DL have escalated rapidly. This increase in computational power became critical to enhancing performance. The authors raised concerns that if the present development persists, these computational criteria will soon become prohibitive both technically and economically. The study indicated that DL development would be restricted by its evaluation metrics and that the ML society would either improve the performance of DL significantly or switch to more efficient ML techniques.

On the other hand, ML has achieved equally significant results. It was effectively used to solve the problems related to clustering, regression, classification, or data dimensionality reduction [20]. ML has demonstrated its tremendous capabilities in a variety of ways. ML was used to develop several useful systems such as the Go-playing system [21], cars with the self-driving ability [22], and image classification [23]. As a result, various aspects of our everyday life are driven by ML algorithms, such as image and speech recognition [24,25], website searching [26], fraud detection [27], email segregation [28], credit count [29], and many more. Data obtained from a computer by different sensors under varying operating conditions are the basis of ML-based fault detection and diagnosis systems. The output of the sensors is generally time series. Features are normally derived using an analysis scheme based on time, frequency, or time-frequency domain analysis. The techniques for studying the frequency domain encompass envelope analysis [30] and high-order spectral analysis [31]. Whereas, techniques for the time-domain analysis include root medium square, high-order statistics, and short-speed impulse [32,33]. Time-frequency domain analysis techniques include Hilbert–Hung, short-time Fourier, and wavelet transforms [34,35]. ML’s key challenge is the tedious and time-consuming process of manually extracting features that require expert knowledge. ML-based classifier might be less accurate than DL without proper discriminant feature extraction and selection. However, if the extraction and selection of the features are performed correctly with knowledge about the type of input data being utilized, greater classification accuracy can be achieved.

Subsequently, several methods for fault detection and diagnoses, such as vibration analysis [36,37,38], electromagnetic field monitoring [39], MCSA analysis, chemical analysis, infrared signal analysis [40,41,42,43], and partial release measurement, have been examined. These fault detection and diagnosis methods are specifically limited to detect the electrical and mechanical faults related only to the electric motor. In an industrial environment, the robots use electric motors coupled with some mechanical parts, such as rotate vector (RV) reducer. This is an additional mechanical part rather than an integral motor part, conjoined with the electric motor to increase or decrease the speed of rotation (rpm). The performance of these reducers degrade over time, and cause the robotic system to work less efficiently with greater power consumption. Normally this happens due to damaged gears inside of the reducer caused by misaligned or cracked teeth. Previously, this type of reducer fault was detected through different procedures, among which the most common use vibration signal analysis, ferrography, or acoustic emission analysis. While vibration analysis has frequently been used, it requires the use of costly vibration sensors. Furthermore, it is difficult to place and install sensors in specific locations to record the vibration signals. The surrounding environment can also create noise, rendering the sensor readings inaccurate. As an alternative, MCSA has several advantages over vibration analysis. MCSA uses the motor control unit’s embedded current signal, requiring no additional sensors, which results in low cost, and a less complex system. In addition, the current signals are unique, and are not easily affected by the surrounding working environment. The problem with implementing MCSA for reducer fault detection and diagnosis is that the reducer is not considered an integral part of the electric motor, making it hard to implement such a technique to distinguish a faulty state. MCSA is typically used to detect faults related to an electric motor. However, in this work we utilize the classification abilities of ML-based classifiers, and present a holistic feature selection and feature extraction approach that is integrated with MCSA, to detect and diagnose the faults related to the RV reducer for a component-level PHM. Using the proposed methodology, the complexity involved in handling vibration signals for such type of fault will be eliminated, and faults can be detected using only the three-phase electric current of the motor. When it comes to real-time implementation, the fault detection system will have a fast response with less computational time (compared to DL). The sensing components will be enormously reduced, which will effectively decrease the cost of the overall system. Besides, we analyze the procedures involved in developing such kinds of systems in real-time, and present practical results obtained using an industrial robot. 

This paper is divided into the following sections: Section 1 provides an introduction, while Section 2 describes the materials and methods used. Section 3 then presents the results and discussion, and finally, Section 4 concludes the study.

## 2. Materials and Methods

**Figure 2:**
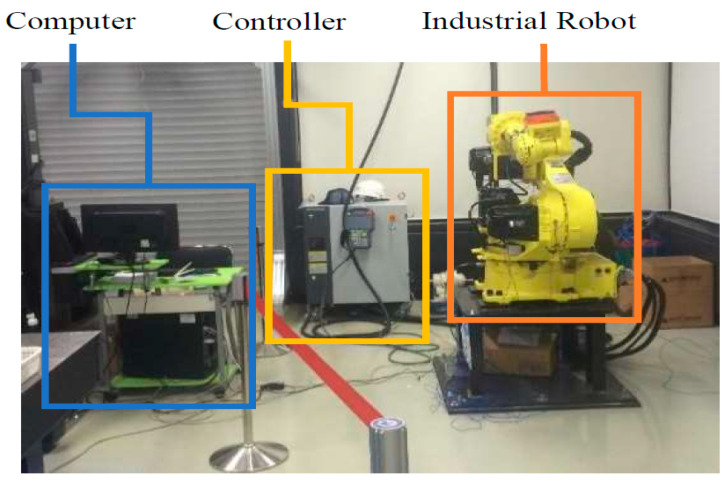
Components of the experimental test bench.

**Figure 3:**
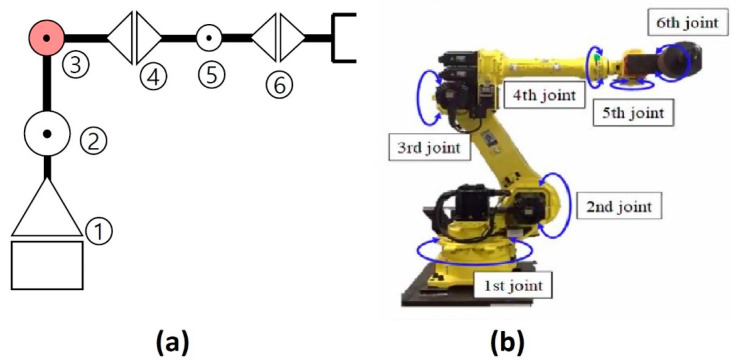
(**a**) Free-body diagram, and (**b**) actual Image of the Hyundai Robot YS080.

The experimental test bench used in this study has three key components: (1) the Hyundai Robot YS080, (2) a controller device, and (3) a personal computer (PC). Figure 2 shows the components of the experimental test bench. The proposed method is implemented on an industrial robot developed by Hyundai Robotics. The robot model is YS080 and has a maximum payload capacity of 80 kgf. The robot consists of six joints or axes. Each axis is equipped with an electric motor of different specifications. With the help of electromechanical couplings, the robot can rotate 360 degrees along each axis. Consequently, at each axis, the electric motors are coupled with reducers to increase or decrease rotational speed. Figure 3 shows (a) the basic free-body diagram, and (b) an actual image of the Hyundai Robot, YS080, identifying the six joints.

The three-phase servo motors are mounted on each axis. The motors with different specifications are mounted based on the amount of mechanical load that each axis holds. Axes 1, 2, and 3 are composed of motors with electrical power higher than those of axes 4, 5, and 6. The motors have different rotational speeds and frequencies. Table 1 summarizes the specifications of the electric motors on each axis. These electric motors are controlled by a controller device that is comprised of three-phase servo motor drivers. The controller is further connected to a personal computer (PC) to send commands to the robot to execute different types of tasks.

### 2.1. The Architecture of the Proposed Methodology

**Figure 4:**
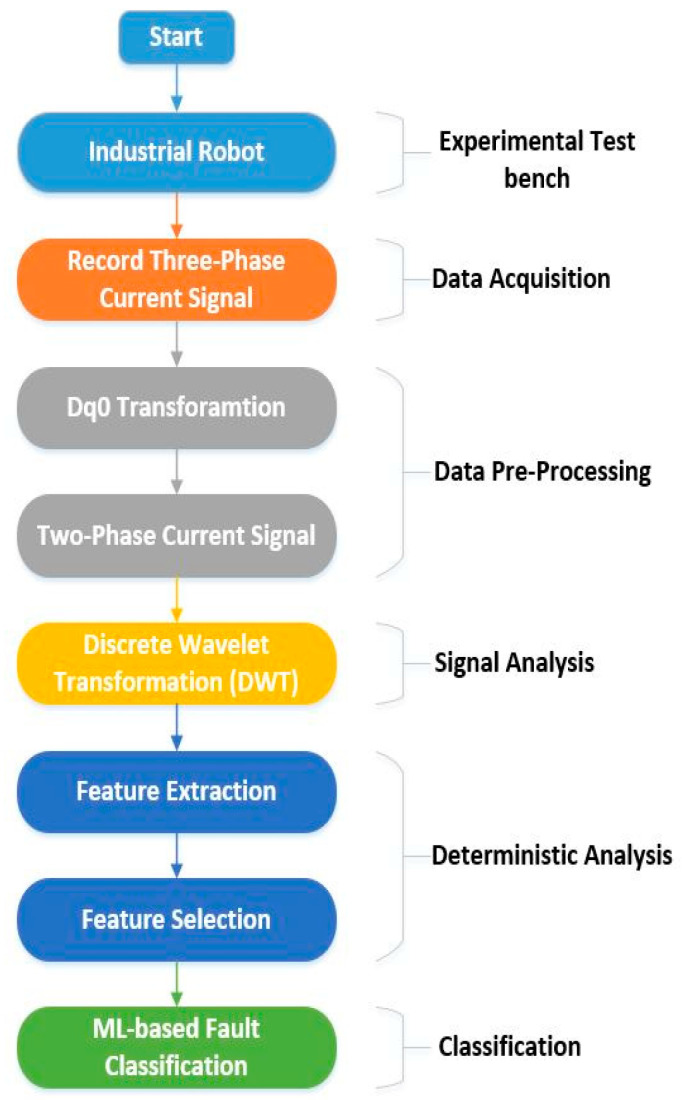
A flowchart of the proposed methodology.

Figure 4 shows the basic flow chart of the proposed fault detection and diagnosis methodology for the RV reducer based on MCSA with ML-based classification:

The proposed method is divided into the following steps:

1. Data acquisition
2. Data-preprocessing
3. Signal analysis
4. Deterministic analysis
5. Classification

To implement the proposed method, an experimental test bench based on an industrial robot was used. Initially, the data are recorded employing current sensors installed at each of the electric motors’ three-phases. Data are recorded for each motor installed at a specific point of the industrial robot. The current signals data in three-phase is pre-processed, and data dimension reduction is performed to compress the data. The data dimension is reduced using the DQ0 transformation. The DQ0 transformation converts the three-phase current signals to two-phase current signals, without losing any useful information. Using discrete wavelet transform (DWT), the two-phase current signals are further analyzed in the time-frequency domain. DWT breaks down the signals into wavelets, which are further used to extract features. Several features are extracted from the wavelets based on wavelet characteristics and statistics. Then, these features are analyzed using feature selection algorithms to select the most prominent and deterministic features. Upon determining the prominent features among the extracted features, ML-based classifiers are trained to categorize among the various classes of faults. A detailed description of each step is given in the subsequent subsections.

#### 2.1.1. Data Acquisition

**Figure 5:**
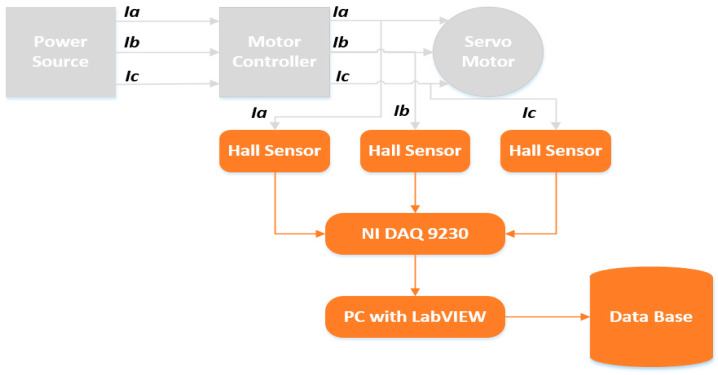
Basic block diagram of the data acquisition process for one axis motor.

For MCSA, the motor current signals were recorded for each of the three phases of the electric motor using current sensors. Figure 5 shows the basic block diagram of the data acquisition process for one axis motor. The current sensors used for this purpose were the Hall Effect Base Linear Current Sensors WCS6800. The current sensors are installed on each phase of the electric motors, i.e., 18 current sensors in total to record 18 current signals for 6 electric motors. The current signals for each axis motor are recoded using NI DAQ 9230 modules. This data acquisition module sends the recorded data to a PC with LabView installed on it. The received signals are analyzed, and a final database comprising of the signal information for each axis motor’s three-phase current signals is formed. 

**Figure 6:**
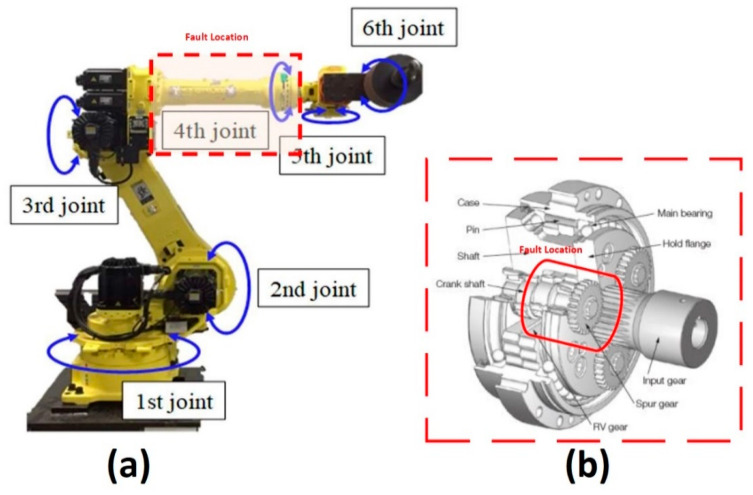
Location of the fault: (**a**) Hyundai Robot, and (**b**) Detailed conceptual view.

**Figure 7:**
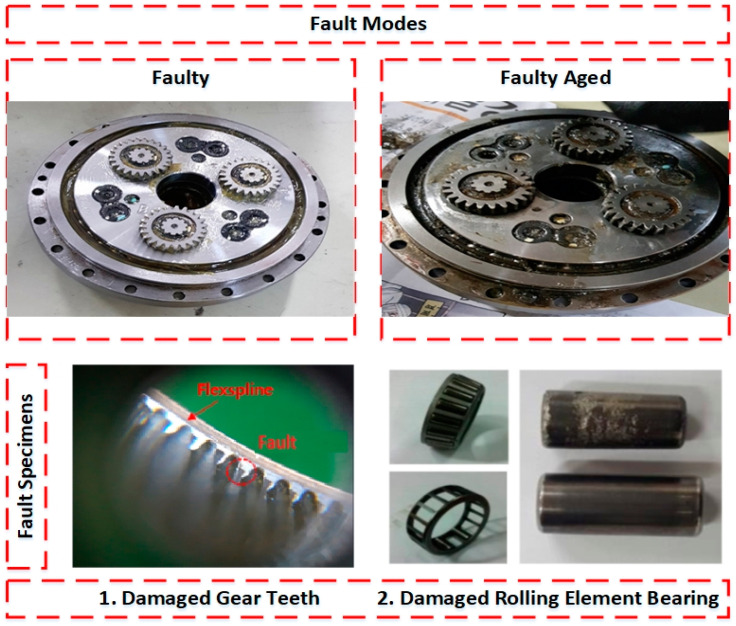
Fault modes and an example of a fault specimens.

**Figure 8:**
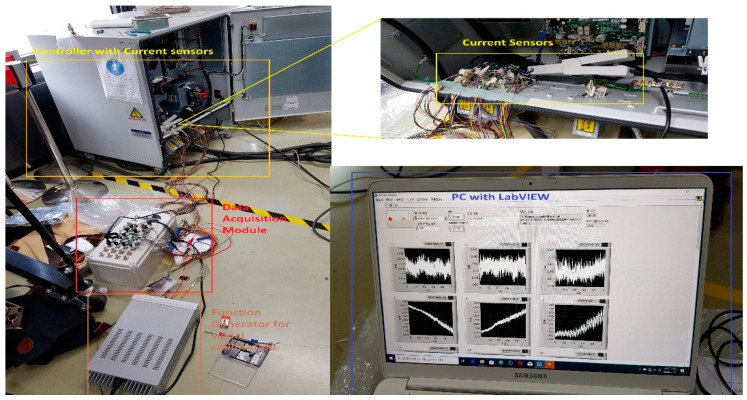
The equipment used in the data acquisition process.

The data are recorded simultaneously for each motor under different fault scenarios. In the first scenario, an RV reducer eccentric bearing fault was inserted in the reducer coupled with the 4th axis motor. In the second scenario, the fault was inserted by replacing the RV reducer with a deteriorated one. The data were recorded for a total of three classes: normal, faulty (RV reducer eccentric bearing fault), and faulty_aged (RV reducer aging fault). Figure 6 shows the location of the fault in the Hyundai Robot with a detailed conceptual view. Figure 7 shows the fault modes with an example of a fault specimen. The robot was made to operate in all directions along each axis of rotation for several cycles. One cycle refers to the completion of one range of motion along one axis. The data were recorded for 10 cycles for each axis. Subsequently, the motors were operated at different speed profiles ranging (10 to 100) % of the rated speed to observe the effect of the change on the speed of fault detection and diagnosis system. Figure 8 shows the details of the equipment used in the data acquisition process. The data are recorded for each axis motor, even though the fault is just inserted into the RV reducer at axis 4, since due to mechanical coupling, a fault in the one axis might affect the operation and efficiency of the other axis motors.

#### 2.1.2. Data Pre-Processing

**Figure 9:**
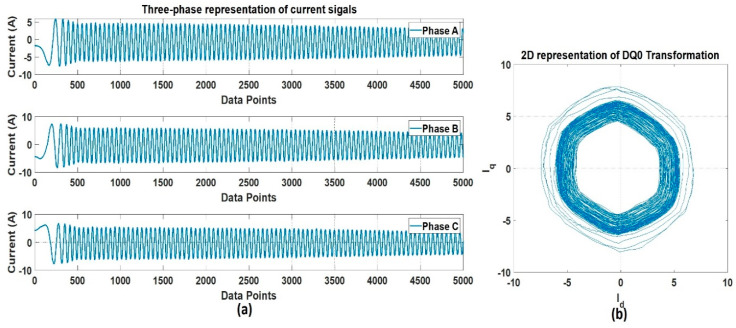
The current signal representation: (**a**) Three-phase, and (**b**) 2D representation of DQ transformation.

**Figure 10:**
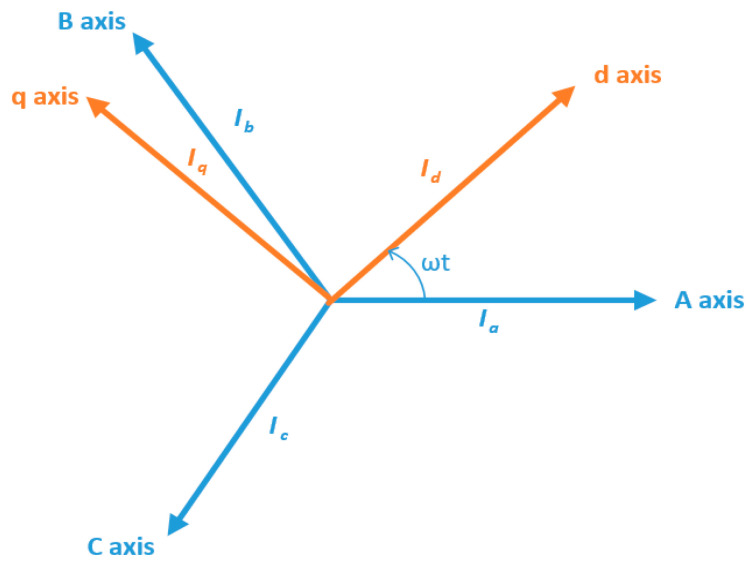
Conceptual representation of three-phase (abc), and DQ0 reference frames.

The recorded data were pre-processed to reduce the dimension in such a way that the useful information from the obtained three-phase current signals would not be missed. The data dimension reduction is performed to make the data compression compact, and more manageable. The DQ0 transformation was used for this purpose. The DQ0 transformation is a well-proven technique for dimensional reduction, converting a three-phase current signal to a DQ0 rotating reference frame. This transformation projects the information from a 3D space to a 2D space, without any loss of information. For the sinusoidal balanced signal, the projected signal describes a circle in the projected plane. This simplifies the frequency estimation, since the circle simply corresponds to the analytic signal. The transformation preserves the amplitude of the electrical components (such as voltages and currents), and is widely used in electrical engineering to implement and design the control parameters of an electric motor. Figure 9 shows the signal representation of (a) three-phase, and (b) 2D, DQ transformation. Figure 10 shows the conceptual representation of the three-phase (abc) and (DQ0) reference frames.

There are two types of DQ0 transformations: cosine-based, and sine-based. Cosine-based transformation aligns the rotating DQ frame with A-axis at *t* = 0, and results in *d* = 0, *q* = −1, *zero* = 0. Sine-based transformation aligns the rotating DQ frame 90 degrees behind A-axis at *t* = 0, and results in *d* = 1, *q* = 0, *zero* = 0. Both of these transformations differ in the aligned reference axis. In the cosine-based transformation, the d-axis is aligned with the A-axis, whereas in the sine-based transformation, the q-axis is aligned with the reference A-axis. When it comes to data dimensionality reduction, the results for each case are similar. Therefore, in this work, we utilized the sine-based transformation given in Equation (1):

(1) $$\begin{array}{c} T_{abc-dq}=\sqrt{\frac{2}{3}}\left[\begin{array}{ccc} \sin\omega t & \sin\left(\omega t-\frac{2\pi }{3}\right) & \sin\left(\omega t+\frac{2\pi }{3}\right)\\ \cos\omega t & \cos\left(\omega t-\frac{2\pi }{3}\right) & \cos\left(\omega t+\frac{2\pi }{3}\right)\\ \frac{1}{\sqrt{2}} & \frac{1}{\sqrt{2}} & \frac{1}{\sqrt{2}} \end{array}\right] \end{array}$$

#### 2.1.3. Signal Analysis

**Figure 11:**
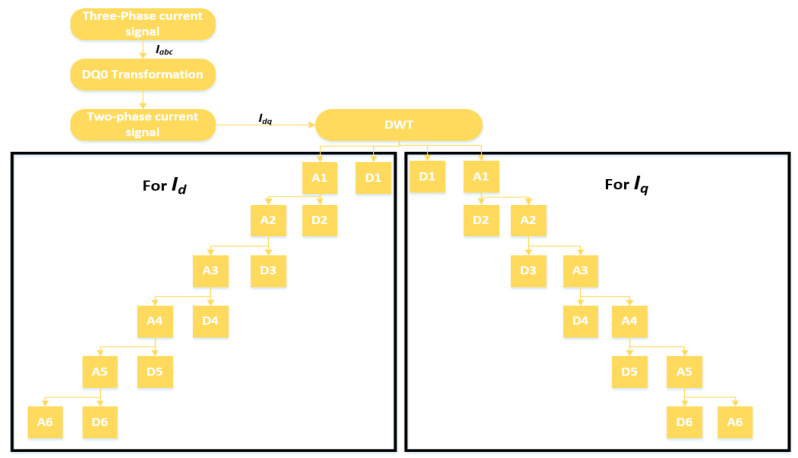
Signal decomposition tree.

The signals extracted from the sensors are stored in a database. Signal analysis is performed to analyze the pattern, and the difference between normal and faulty conditions. Signal analysis is the most important step of any fault detection and diagnosis. Different kinds of analysis schemes are developed for this purpose. These schemes can be categorized into time-domain, frequency-domain, and time-frequency domain analyses. In the time-domain analysis, statistical features summarizing the useful information in the time-domain are extracted from the signal [44,45,46]. On the other hand, frequency-domain analysis is believed to work well in distinguishing some faults with certain characteristics. Fourier transform is the most commonly used tool for frequency-domain signal analysis. It breaks down a time waveform into its frequencies. Fast-Fourier Transform (FFT) is commonly used for the study of time continuum signals. This transform utilizes the spectral frequency analysis scheme. At a certain frequency, the signature of a fault may be a high degree of vibration. When it comes to the processing of non-stationary signals (typically in machine faults), the time, and frequency domain analysis have specific limits. Time-frequency domain analysis, a blend of frequency and time domains, has been developed to address these limitations [47]. A typical method used for this purpose is called the short-time Fourier transform (STFT) [48], which divides the entire waveform signal through short-time windows and Fourier transforms into several segments. Another common approach with a similar aim is wavelet transformation. Wavelet Transform is an analytical method that uses a definition of spectral decomposition by the scaling concept for time-varying or non-static signals. Of the many techniques in the past that have been developed for different signal-processing applications, wavelet theory offers a dedicated and compact framework [49,50]. Multi-resolution signal analysis with a good time- and the frequency-localization mechanism is one of its several features. It is effective for both stationary and nonstationary signal processing. The most commonly used method among the wavelet transform is the discrete wavelet transform (DWT). In numerical and functional analyses, a discrete transform wavelet (DWT) is a transform that discreetly samples the wavelets. In comparison to the other wavelet transforms, a crucial advantage it has over Fourier transforms (frequency-domain analysis) is temporal resolution, capturing both frequency and location information (location in time). Therefore, in this work, we selected time-frequency domain analysis using DWT to analyze the signals recorded under different fault conditions. Figure 11 shows the decomposition tree of the recorded current signals. The three-phase current signals $I_{abc}$ are pre-processed using DQ0 transformation. The output of the Dq0 transformation is a two-phase signal $I_{dq}$ in the d-axis and q-axis. This two-phase signal is decomposed into wavelets using DWT. 

Considering $I_{d}\left[n\right]$ and $I_{q}\left[n\right]$ as the original signal sequence, after the convolution with h and g quadrature mirror filters, the signal sequence is decomposed into the approximation $A_{1}\left[n\right]$ and detail components $D_{1}\left[n\right]$ at level 1. Then, the approximation component $A_{1}\left[n\right]$ is further decomposed into $A_{2}\left[n\right]$ and $D_{2}\left[n\right]$ at the next level. This process is repeated until a desired level of decomposition is achieved. Mathematically, it can be represented by Equations (2)–(5):

(2) $$\begin{array}{c} A_{m}\left[n\right]=\overset{\infty }{\underset{k=-\infty }{\sum }}g\left[n-k\right]I_{d}\left[k\right] \end{array}$$

(3) $$\begin{array}{c} D_{m}\left[n\right]=\overset{\infty }{\underset{k=-\infty }{\sum }}h\left[n-k\right]I_{d}\left[k\right] \end{array}$$

(4) $$\begin{array}{c} A_{m}\left[n\right]=\overset{\infty }{\underset{k=-\infty }{\sum }}g\left[n-k\right]I_{q}\left[k\right] \end{array}$$

(5) $$\begin{array}{c} D_{m}\left[n\right]=\overset{\infty }{\underset{k=-\infty }{\sum }}h\left[n-k\right]I_{q}\left[k\right] \end{array}$$

where, m, n, and k represent the scale of decomposition, sample points, and translation coefficients, respectively. There are several types of the wavelet transform, but among them all, the most widely used series of discrete wavelet transforms was developed by the Belgian mathematician Ingrid Daubechies in 1988. The Daubechies wavelet transform is based on the use of the recurrence relationships to yield increasingly fine and distinct samples of an implicated mother wavelet function [51,52]. In this work, we focus on using 6-level Daubechies wavelets. Levels are selected by observing the decomposition state of the signal at different levels. 

#### 2.1.4. Deterministic Analysis

Upon implementing the DWT on the DQ0 transformed current signals, several features were extracted from the decomposed wavelet signals. The feature extraction and selection is the most important part of any fault detection and diagnosis system. Especially when it comes to the ML, improper feature extraction and selection can cause poor classification accuracy. As mentioned in the introduction section of the manuscript, ML’s key challenge is its tedious process of manual feature extraction. ML-based classifier might be less accurate than DL without proper discriminant feature extraction and selection. However, if the extraction and selection of the features are performed correctly with the knowledge about the type of input data being utilized, greater classification accuracy can be achieved. 

In this work, we adopt an approach where we extract two types of features from the decomposed wavelets of the original current signals. The first type of features are solely based on the wavelet characteristics, and focus on the wavelet domain, whereas the second type of features are extracted based on statistical analysis. The reason for extracting different types of features is that in typical analysis methodologies, either the first type of feature is utilized, or the second type. Also, it is dependent on the type of fault being diagnosed. In the case of RV reducer fault detection and diagnosis, due to the high sensitivity of the fault, the typical feature extraction methodologies fail to provide higher classification accuracy. Therefore, we developed a method where we extract features based on both of the above-mentioned types, and implement deterministic feature selection to choose the most prominent features among them. Doing so provides the power to utilize the properties of wavelet and statistical domain simultaneously in an efficacious way. We name these two types as (1) wavelet specific features, and (2) wavelet-based statistical features. Wavelet specific features extracted from wavelets are the wavelet energy and Shannon wavelet entropy. Wavelet-based statistical features extracted from wavelets are the mean, standard deviation, variance, kurtosis, and skewness. Table 2 and Table 3 define the wavelet specific features and wavelet-based statistical features, respectively.

**Figure 12:**
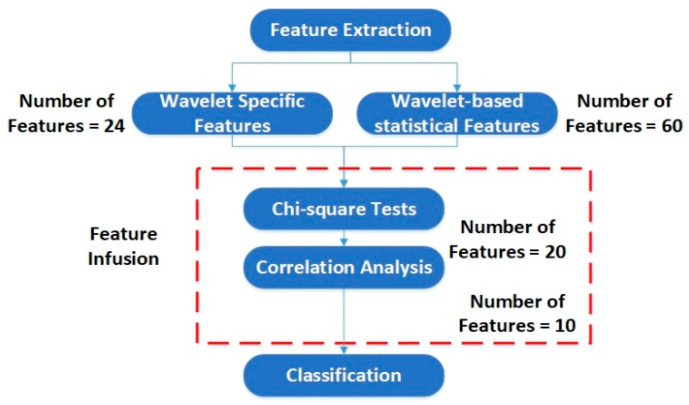
Flowchart of the feature selection scheme.

In Table 2 and Table 3, $D_{i}$ and $A_{i}$ are the detail and approximation components of the $i_{th}$ level decomposed signal, respectively; s is the input signal, and $s_{i}$ is the coefficient of s an an orthonormal basis; n is the value of one observation, and N is the number of observations. We extracted the features from each detail coefficient (D1 to D6) for both the $I_{d}$ and $I_{q}$ components of the current signal. A total of 24 wavelet specific features and 60 wavelet-based statistical features were extracted. Table 4 and Table 5 show the details of the extracted features. These extracted features were further passed through some feature selection algorithm based on the correlation analysis and chi-square tests to reduce the number of features. The features with prominent patterns were distinguished and used for the classification of the faults. Figure 12 shows the flow chart of the feature selection scheme.

## 3. Results and Discussion

**Figure 13:**
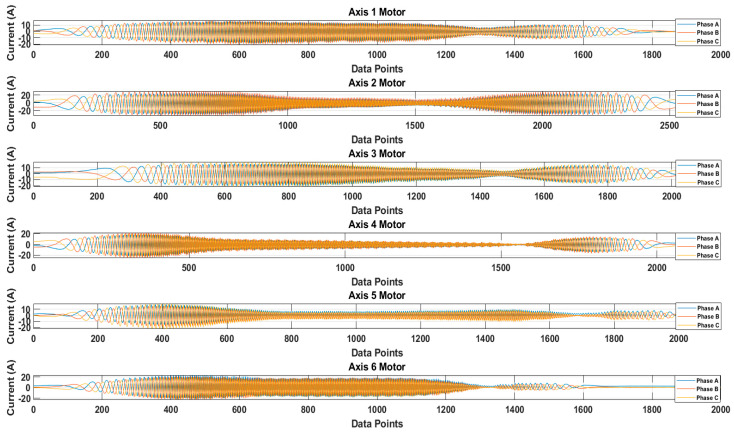
Recorded waveforms of the three-phase current signals for each axis motor.

**Figure 14:**
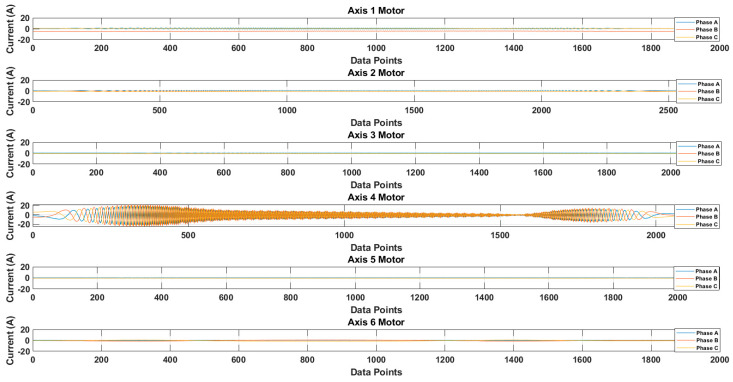
Recorded waveforms for single-axis motor operation.

Figure 13 shows the recorded waveforms of the current signals for each axis. The current signals were recorded by operating each axis motor. The data were recorded for 10 cycles of rotation. For the sake of simplicity, only one cycle’s data are presented. Figure 14 shows the recorded waveform when only the 4th axis motor was operating. 

**Figure 15:**
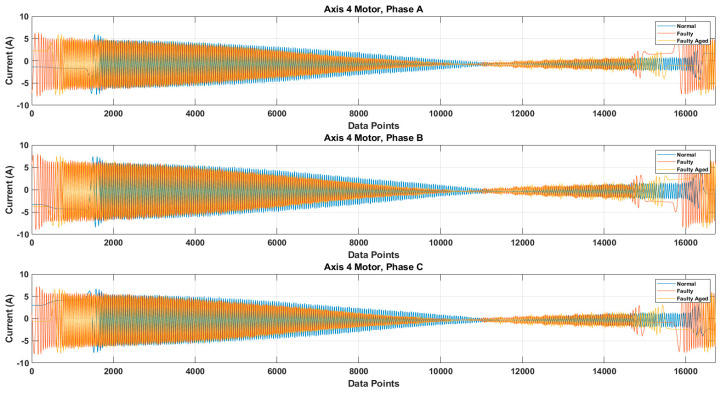
Three-phase current signal for 4th Axis motor under Normal, Faulty, and Faulty_Aged scenario.

**Figure 16:**
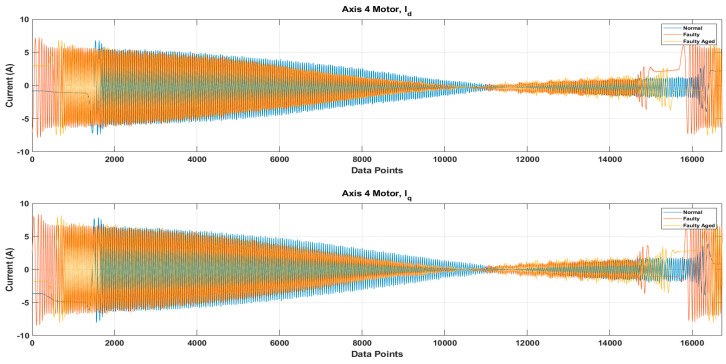
The DQ0 transformed two-phase current signals under Normal, Faulty, and Faulty_Aged scenario.

It is possible that because of the mechanical couplings between each axis of the robot, during the normal and faulty states, the operation of one axis motor might affect the other axis motors, leading to simultaneous current signature analysis. In the simultaneous current signature analysis, the current signals of all motors should be analyzed, regardless of the fault location. This leads to more complex fault detection and diagnosis system, but upon several experimental findings with the results presented in Figure 14, it is clear that for a robotic arm like the Hyundai Robot, the mechanical couplings along the robotic arm do not affect the current signals of another motor. This gives the possibility of focusing on only single-axis current signature analysis. In case there is some fault in an axis, only that axis current signature analysis would be enough to distinguish the fault, without the worry of the mechanical relation between the different axes of the robot. Based on this and the fault location (RV reducer of the 4th axis), we focused only on the 4th axis current signature analysis. We recorded the current signals for the 4th axis motor under three different fault scenarios: normal, faulty (RV reducer eccentric bearing fault), and faulty_aged (RV reducer aging fault). Figure 15 shows the three-phase current signal for the 4th axis motor under normal, faulty, and faulty_aged scenarios. These three-phase current signals were converted to two-phase using DQ0 transformation to achieve the reduced data dimensions. Figure 16 shows the DQ0 transformed two-phase current signals for the 4th axis motor under each fault scenario. 

**Figure 17:**
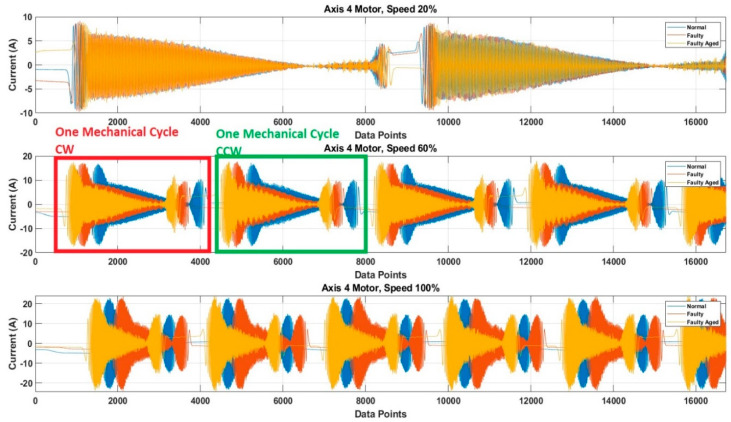
The DQ0 transformed current signal for each fault scenario under (20, 60, and 100) %.

The RV reducer fault is a mechanical fault, and if all the operating parameters, such as speed, frequency, amplitude, and signal shape, are not considered properly, the use of MCSA for the detection of such kind of fault can be a complicated task. Normally, when it comes to the detection of electrical and electronic faults related to electric motors and their controller devices, the response of the current signal shows a similar pattern under different operating parameters, due to which it becomes easy to detect the fault by just observing the current signal response with selected parameters [53,54,55]. However, in the case of RV reducer, due to the fault nature and electromechanical relation between the motor and reducer, it would be hard to achieve higher accuracy if a fault detection and diagnosis system is not developed with consideration of all the operating parameters. Among these parameters, the speed of the rotation of the motor is the most important parameter, due to its direct relation to the reducer device. Considering this factor, we recorded the data for different operating speeds of the motor, to observe the effect of speed change in each fault scenario. We selected a speed profile where we recorded the signal response of the motor at the speed range (10 to 100) % of the rated speed. This gave us an overall view of the faults. We present the current signal response for the speed of 20%, 60%, and 100%. Figure 17 shows the DQ0 transformed current signal for each fault scenario under 20%, 60%, and 100%. It can be observed that with the increase in the rotation speed of the motor (rpm), the amplitude, frequency, and mechanical rotational speed of the robot along the axis also increase. In Figure 13, Figure 14, Figure 15 and Figure 16, the rpm was 10% of the rated speed. In Figure 17, one mechanical cycle corresponds to the mechanical rotation of the robot along each axis. The robot moves clockwise (CW) to complete one cycle, and returns to its starting position by moving counterclockwise (CCW). With the increase in the rpm of the motor, the mechanical cycles of the robot also increase.

After data acquisition and DQ0 transformation, we implemented DWT on components $I_{d}$ and $I_{q}$ of the current signal for all fault scenarios. The selection parameters, including the sampling frequency and the number of samples, should be carefully selected to achieve the right resolution for wavelet analysis. Few restrictions have been taken into account for this, including (1) signal bandwidth, (2) wavelet spectral band decomposition, (3) frequency resolution, and (4) acceptable level of decomposition. The use of the Shannon theorem provides a minimum sampling frequency of 1000 Hz. Equation (6) gives the total number of samples, Ns, required for an already given resolution R [20,21,22].

(6) $$\begin{array}{c} Ns=\frac{f_{s}}{R} \end{array}$$

**Figure 18:**
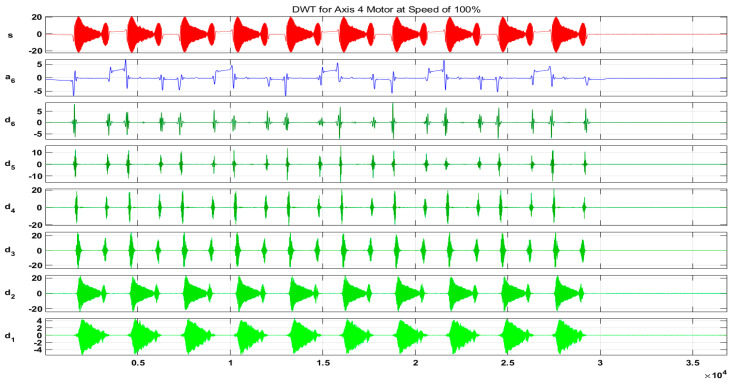
Output results of the DWT for Id component of the current signal at a speed of 100%.

**Figure 19:**
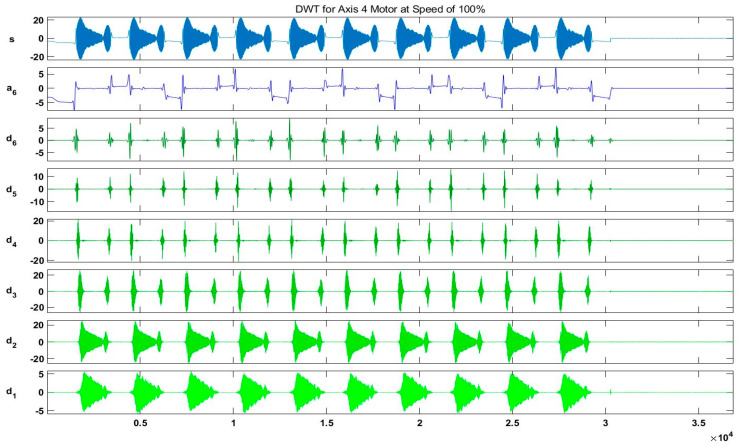
Output results of the DWT for Iq component of the current signal at a speed of 100%.

**Figure 20:**
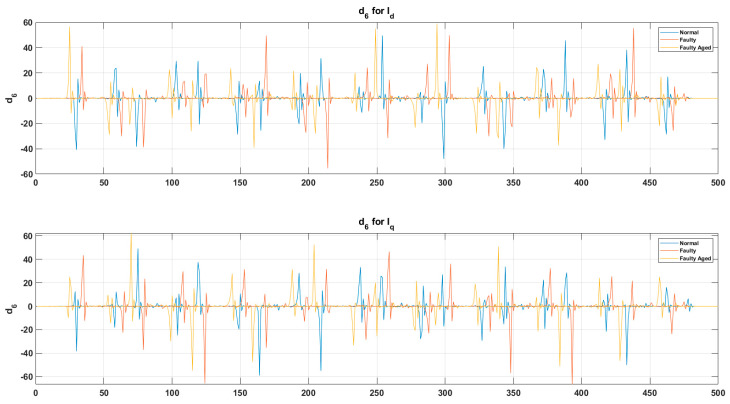
Detailed view of the D6 decomposed wavelet coefficient for each fault scenario.

In our case, for the resolution of R=0.1 Hz, we chose a sampling frequency $f_{s}=12.8\;\mathrm{kHz}$. Hence, Ns=128,000 samples were acquired. Figure 18 and Figure 19 show the output results of the DWT for $I_{d}$ and $I_{q}$ components of the current signal at a speed of 100% without any fault, whereas Figure 20 shows a detailed view of the D6 decomposed wavelet coefficient for each fault scenario. In these figures, s is the original signal, a is the approximation coefficient, and d is the detail coefficient extracted by the decomposition of the original current signal using six-level Daubechies wavelets.

**Figure 21:**
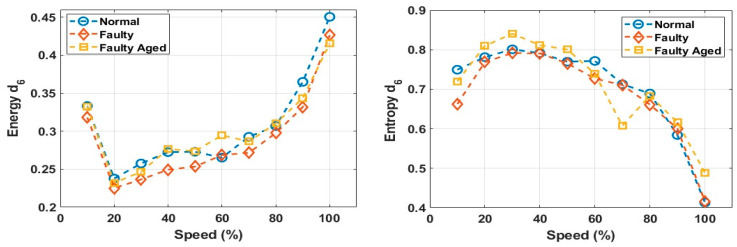
Response of the wavelet specific features at different speeds.

**Figure 22:**
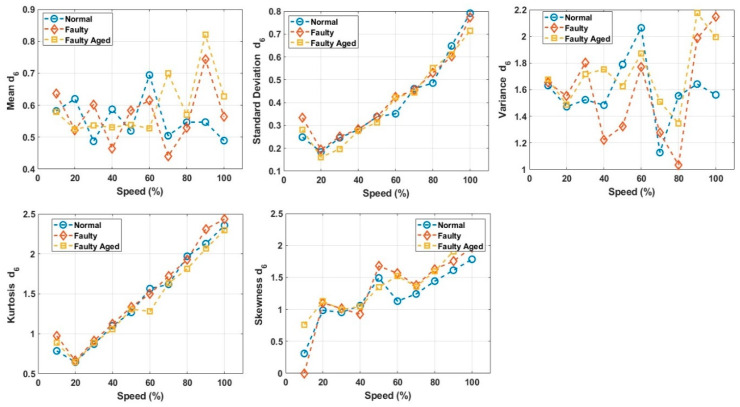
Response of the wavelet-based statistical features at different speeds.

**Figure 23:**
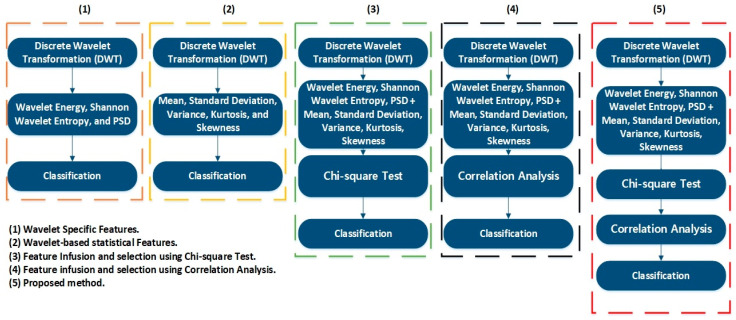
Flowchart of the Case Study.

These decomposed wavelets were utilized for further deterministic analysis. Several features presented in Table 3 and Table 4 were extracted from each decomposed wavelet coefficient. Figure 21 shows the response of the wavelet specific features, while Figure 22 shows the response of the wavelet-based statistical features extracted at different speeds of rotation of the motor. The presented results are for the detailed coefficient D6. It is apparent that among these features, some features show a very clear difference between the fault categories at some certain speed, though devoid of an overall constant pattern for all of the speed profiles, due to the natural operating phenomenon of the motor. During the operation of the motor, the amount of current flowing through the windings increases or decreases, depending on the rotation speed. This causes analytical uncertainties for classification systems that rely on the MCSA. The classification systems function by autonomously discovering the patterns in data. In the cases of features with less prominent patterns, the accuracy of the systems falls drastically. In particular, in RV reducer fault detection and diagnosis, it is hard to find some prominent patterns among several features. For this, we utilized the feature selection scheme presented in Figure 12 for prominent feature selection, citing some case studies for each type of feature and classification accuracy. We used four main types of ML-based algorithms for classification, namely (1) linear discriminant analysis (LDA), (2) fine tree (FT), (3) naïve Bayes (NB), and (4) support vector machine (SVM). Figure 23 shows the flowchart of the case study for this paper.

### 3.1. Case 1: Wavelet Specific Features

**Figure 24:**
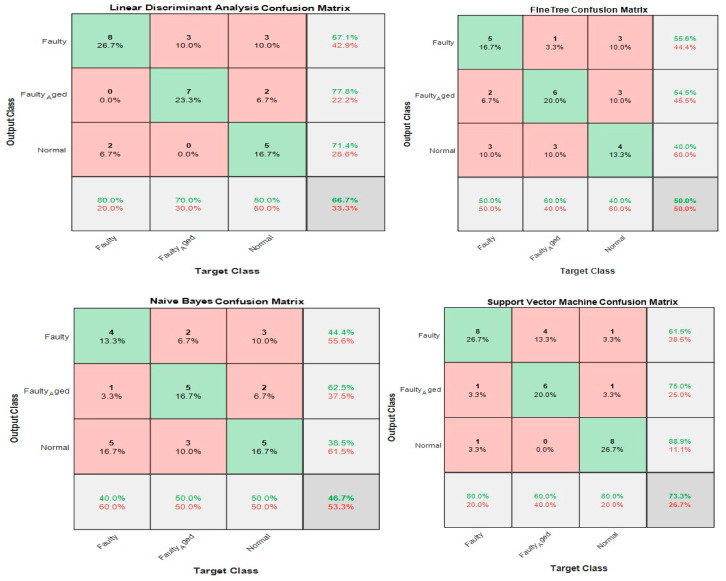
Classification results for Case 1: Wavelet Specific Features.

In this case, the 24 wavelet specific features presented in Table 4 were used to train the aforementioned four types of classifiers. We implemented five-fold cross-validation in this work to prevent the model overfitting. The available data were segregated into five disarticulated folds. Among these five folds, four folds were utilized as training samples and one-fold as a testing sample under every training iteration. Every data sample was utilized precisely once as a testing sample. On all folds, the average test error is determined. Using this training and validation scheme, the predictive precision and accuracy of the final model trained with all the data are measured. Figure 24 shows the classification results for the wavelet specific features. The results are presented in the form of a confusion matrix. The rows refer to the predicted output class, whereas columns refer to the true target class. The diagonal cells refer to the classes that are accurately classified. The off-diagonal cells refer to the classes that are inaccurately classified. Each cell presents the number of observations and their percentage. The far-right column in the confusion matrix presents the percentages of all the observations predicted for each class, classified correctly and incorrectly. These matrices are called the precision and false discovery rate. The overall accuracy of the classifier is given in the cell at the bottom right. The accuracy is calculated using Equation (7):

(7) $$\begin{array}{c} Accuracy=\frac{TP}{TP+FN} \end{array}$$

where TP is true positive and FN is false negative. In this case, the maximum accuracy of 73.3% was achieved for the SVM, followed by 66.7%, 50%, and 46.7% for LDA, FT, and NB, respectively.

### 3.2. Case 2: Wavelet-Based Statistical Features

**Figure 25:**
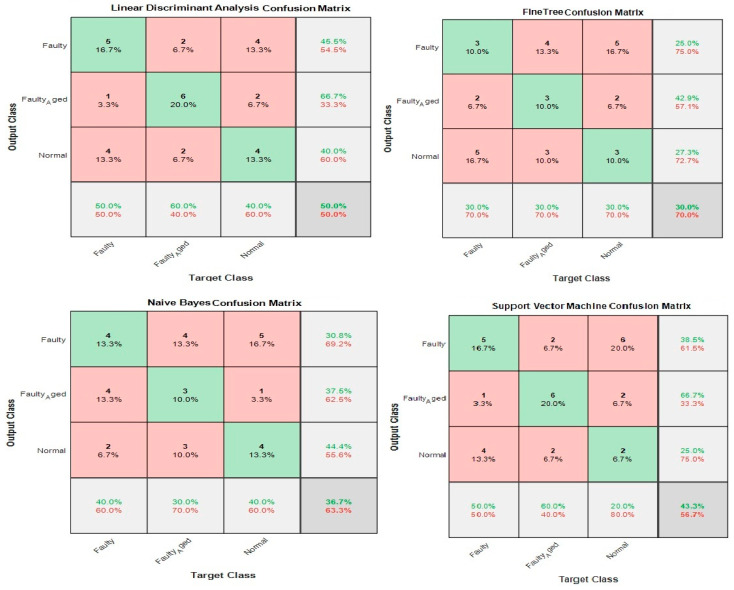
Classification results for Case 2: Wavelet-based Statistical Features.

Table 5 shows the 60 features we used in this case, while Figure 25 shows the classification results for the wavelet-based statistical features. The number of features used is based only on the statistics (Table 5). The maximum accuracy of 50% was achieved for the LDA, followed by (43.3%, 36.7%, and 30.0% for SVM, NB, and FT, respectively. The accuracy achieved was very poor, and this is because the statistical features, such as mean, standard deviation, variance, kurtosis, and skewness, somehow relate to one another in statistical characteristics. While using these kinds of features, there are more chances for the classifier to become confused among several parameters. Regardless of this fact, these features were not ignored and eliminated from performing any kind of classifications; rather, we implemented a feature selection and infusion scheme to distinguish meaningful features, which will be discussed in cases 3, 4, and 5. 

### 3.3. Case 3: Feature Infusion and Selection Using Chi-Square Test

In this case, we utilized the typically used statistical features selection algorithm, univariate feature ranking for classification, using chi-square tests *fscchi2* for the selection of prominent features among the features presented in Table 4 and Table 5. *fscchi2* explores if all predictor variables are independent of response variables, by utilizing individual chi-square tests. 

The chi-square test of independence decides if there is a relation among categorical variables (i.e., if the variables are independent, or associated). A nonparametric test is commonly used to test the statistical independence or relation among two or more categorical variables. This test uses a contingency table to evaluate the data. A contingency table is a structure in which data are categorized according to two categorical variables. It is also known as a cross-tabulation or a two-way table. For one variable, the categories appear in rows, while for the other variable, they appear in columns. Two or more categories should be used with each variable. Each cell in the table represents the total number of cases for a certain category pair. Equation (8) gives the mathematical representation of the *fscchi2:*

(8) $$\begin{array}{c} X^{2}=\overset{R}{\underset{i=1}{\sum }}\overset{C}{\underset{J=1}{\sum }}\frac{{\left(o_{ij}-e_{ij}\right)}^{2}}{e_{ij}} \end{array}$$

where $o_{ij}$ are the observed cell count in the ith row and jth column of the table, and $e_{ij}$ is the expected cell count in the ith row and jth column of the table. This can be calculated as in Equation (9):

(9) $$\begin{array}{c} e_{ij}=\frac{row\;i\;total*col\;j\;total}{grand\;total} \end{array}$$

**Figure 26:**
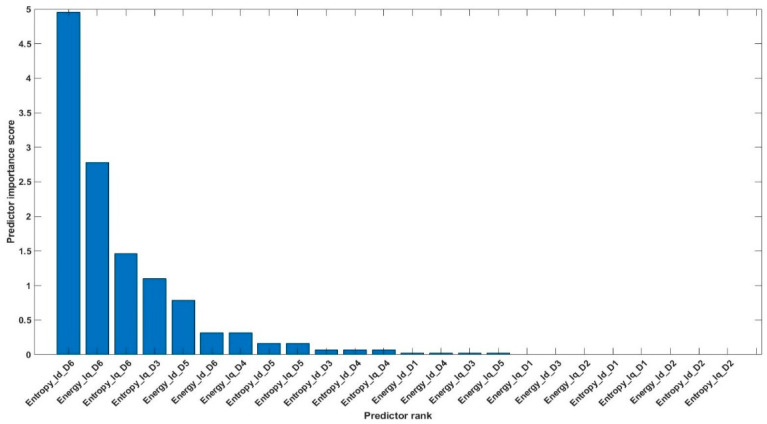
Example of categorization of features based on importance score.

**Figure 27:**
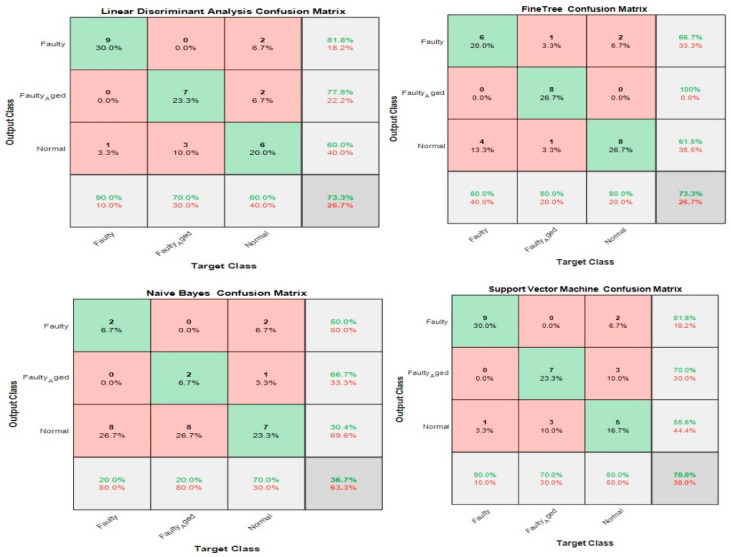
Classification results for Case 3: Feature infusion and selection using Chi-Square Test.

Using the above equations, the number of features was reduced from 84 to 20 (combined features of Table 4 and Table 5). The 20 most valuable features with high importance scores were selected, and classification was performed. Figure 26 shows an example of the categorization of features based on importance. Table 6 gives the details of the 20 most prominent features selected with high importance score. Figure 27 shows the classification results for feature infusion and selection using chi-square test. The maximum accuracy of 73.3% was achieved for the LDA and FT, followed by 70% and 36.7% for SVM and NB, respectively.

### 3.4. Case 4: Feature Infusion and Selection Using Correlation Analysis

Only categorical variables can be compared in the chi-square independence test. It cannot equate continuous variables or continuous variables with categorical variables. Moreover, it assesses only the associations between categorical variables, and cannot provide any inferences about causation. This can be observed also by the accuracy results achieved in the previous case (case 3). 

**Figure 28:**
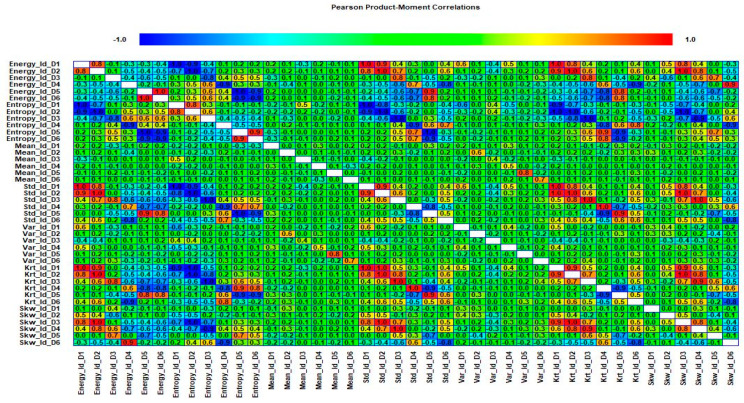
Case 4: An example of one of the correlograms used to select features.

**Figure 29:**
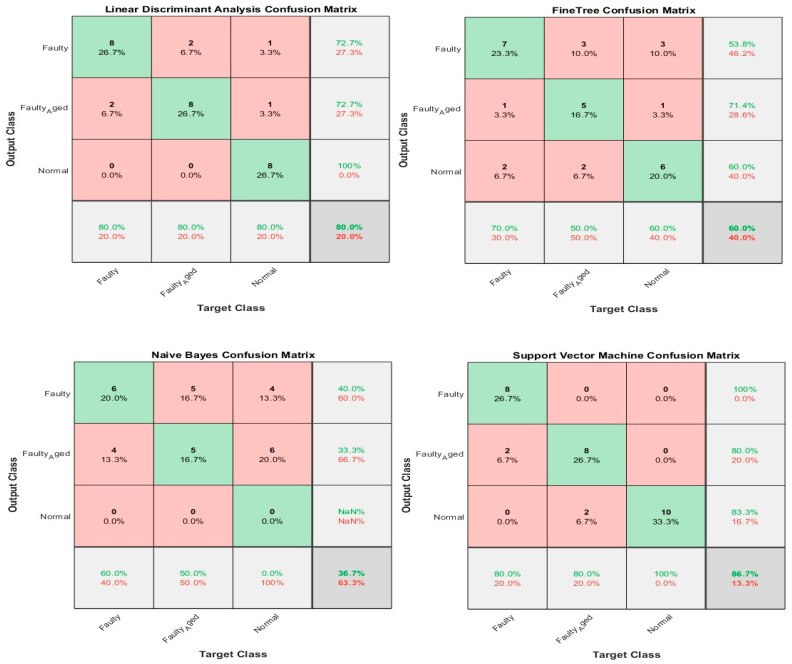
Classification results for Case 4: Feature infusion and selection using Correlation Analysis.

In this case, to evaluate the difference in performance between the chi-square test and correlation analysis, we utilized the correlation analysis, rather than chi-square tests, to select the prominent features from both the wavelet specific and wavelet-based statistical features. The correlation analysis is used to determine the correlation between two variables. These variables can be two independent or a dependent and independent variable. We measure the Pearson product moment coefficient of correlation, in particular, as a sample correlation. The sample correlation coefficient is represented by *ρ* and its values range from −1 to +1. There can be a positive or negative correlation between the two variables. The positive correlation means that the variables are highly correlated to each other and vice versa for the negative correlation. The sign of the coefficient of correlation shows the orientation of the relationship. The magnitude of the coefficient of correlation shows the strength of the relationship. Mathematically, it is given in Equation (10):

(10) $$\begin{array}{c} \rho_{xy}=\frac{\sum_{i=1}^{n}\left(x_{i}-\bar{x}\right)\left(y_{i}-\bar{y}\right)}{\sqrt{\sum_{i=1}^{n}{\left(x_{i}-\bar{x}\right)}^{2}}\sqrt{\sum_{i=1}^{n}{\left(y_{i}-y\right)}^{2}}} \end{array}$$

where n is the sample size, $x_{i},\;y_{i}$ are the individual sample points indexed with i, and $\bar{x}=\frac{1}{n}\sum_{i=1}^{n}x_{i}$ is the sample mean. We selected the 20 most prominent features presented in Table 7. These features were carefully selected by analyzing the correlogram. Figure 28 shows an example of one of the correlograms used to select features. The features that had the lowest values for the correlation coefficients were selected. The features with a higher correlation coefficient value of more than 90% mean that these features show similarities, and can be neglected, to avoid confusion in the training and testing of ML-based classifiers. Figure 29 shows the classification results for feature infusion and selection using chi-square test. The maximum accuracy of 86.7% was achieved for the SVM, followed by 80%, 60%, and 36.7% for LDA, FT, and NB, respectively.

### 3.5. Case 5: Proposed Feature Infusion Method

The correlation analysis performed well in the process of feature selection then chi-square tests, but the accuracy achieved with a high number of features was still low. Generally, for ML-based classification, the number of features is indirectly proportional to the number of observations (Equation (11)):

(11) $$\begin{array}{c} Number\;of\;Features=\frac{1}{Number\;of\;Observations} \end{array}$$

**Figure 30:**
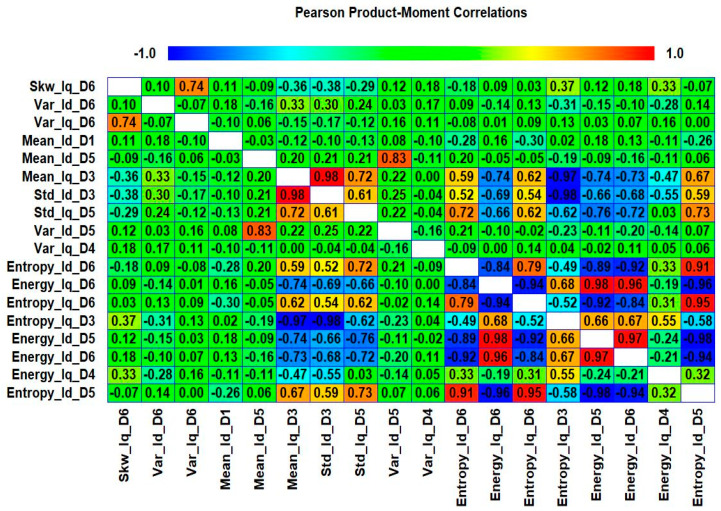
Case 5: The correlogram used to select features.

**Figure 31:**
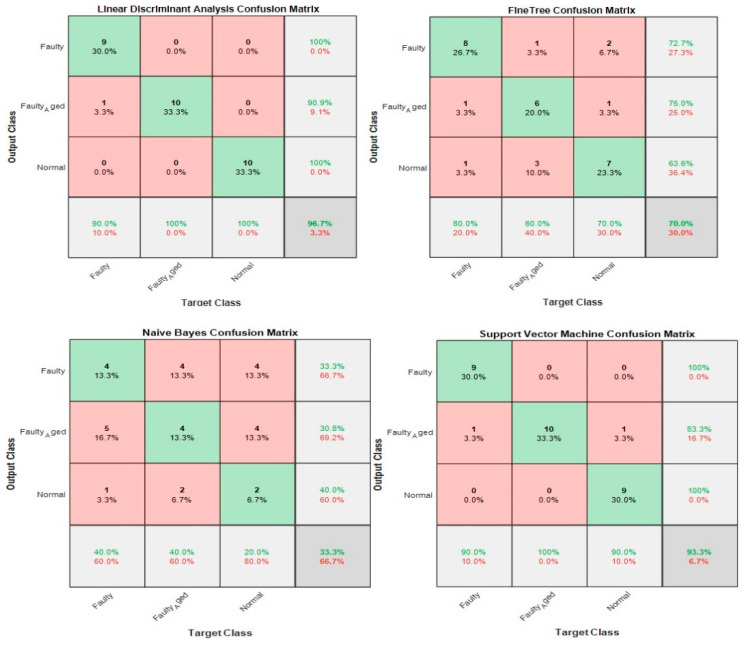
Classification results for Case 5: The Proposed Feature Infusion Method.

The less the number of observations, the more the number of features. In our case, the total number of observations for each class is 10. A lesser number of observations requires more features to achieve higher classification accuracy. On the other hand, a high number of features might also confuse the classifier among several classes, and a good classification accuracy might still not be achieved. Therefore, we combined both chi-square tests and correlation analysis to obtain a balance between the number of features and observations for higher classification accuracy. Figure 23 shows that the proposed method works in two steps of feature selection. First, the chi-square tests were performed to get the importance score of all the features; and among these features, the 20 most prominent features were selected. These selected features were further analyzed, using correlation analysis. Figure 30 shows the correlogram of these features. From these 20 features, we selected the 10 features with a correlation of less than 80%. Table 8 presents these 10 features. Figure 31 shows the classification results for the proposed feature infusion method. The maximum accuracy of 96.7% was achieved for the LDA, followed by 93.3%, 70%, and 33.3% for SVM, FT, and NB, respectively. 

### 3.6. Comparative Study for Different Cases and Other Methods

Table 9 compares the results among different cases. The results in Table 9 prove that the proposed method works well in detecting and diagnosing the RV reducer fault with high accuracy and a lesser number of features. The average accuracy score calculated (Equation (12)) for the proposed method is 73.3, which is much higher than the score of all the other feature selection and infusion cases. Notice that not all the selected classifiers are suitable for fault diagnosis as different classifiers works based on different algorithms. The choice of classifiers can be made based on the type of problem. In our case, the NB performs poorly in the classification of faults because NB works on an algorithm based on Bayes’ theorem. Whereas the LDA shows the best case, and its because LDA is a commonly used multi-variate classification method that aims to find a linear combination of features for class separation which suits perfectly for the type of problem and data we had to deal with in this work. The rightmost side of Table 9 presents the classifier’s average performance score (Equation (13)), based on each case. Among the four classifiers, the LDA and SVM performed better than the FT and NB.

(12) $$\begin{array}{c} Average\;Accuracy\;Score=\frac{\sum_{i=1}^{N}Accuracy}{N} \end{array}$$

(13) $$\begin{array}{c} Classifier\;Average\;Performance\;Score=\frac{\sum_{j=1}^{K}Accuracy}{K} \end{array}$$

In Equations (12) and (13), N is the total number of classifiers and is given as N=1 (LDA),2 (FT),3 (NB),4 (SVM), while K is the total number of cases, and is given as K=1, 2, 3, 4, 5. 

The comparison results among different methods used to detect and diagnose faults are presented in the Table 10. We present a comparison among different techniques and methodologies related to chi-square features [56], discrete wavelet transform with ANN [57], statistically locally linear embedding with SVM [58]. Viney et al. [56], proposed methods where chi-square features were used to classify the faults. Different classifiers with a different number of features were used for this purpose. The accuracy achieved was different for the different number of features. For eight features, the accuracy was 93.33% but for seven features the accuracy reduced to 92%. Just reducing one feature reduced the accuracy by 1%. Compared to our proposed methodology, we presented a consolidated approach for feature extraction and selection giving us the advantage to reduce the number of features and achieve higher classification accuracy at the same time. On the other hand, Konar et al. [57], used DWT with an artificial neural network (ANN) as a classifier. The accuracy achieved for this method was 93.33%. Also, the use of ANN is computationally heavy as compared to other classifying algorithms such as LDA, SVM, etc. Wang et al. [58], utilized statistical locally linear embedding with SVM as a classifier. The accuracy was recorded as 94.07% which is higher than the other methods. The SVM performed well for the classification of the faults but still, the accuracy is not that good enough. Our proposed methodology utilizes the advantages of signal processing and statistical analysis together to form a consolidated approach for the detection and diagnosis of faults. the accuracy achieved was 96.7% which is considerably high than the other methods. The proposed methodology is suitable for the ML-based classifiers and can help reduce the hard work and labor involved in the feature extraction and selection.

## 4. Conclusions

In an electromechanical system, generally, two types of faults can occur. These faults are electrical faults and mechanical faults. The electrical faults can be categorized into further three categories: (1) power source faults, (2) inverter/converter faults, and (3) machine faults (electric motors faults). The mechanical faults can be categorized as (1) faults related to electric machines and (2) faults related to coupled mechanical components. In the case of electrical faults and mechanical faults related to electrical machines, electric current signal is used to find the abnormality in the related components. On the other hand, the faults related to coupled mechanical components are detected using vibration, acoustic emission, or ferrography analysis. This leads to segregated and more complex ways for any fault detection and diagnosis system. Therefore, in this work, we focused on the detection of the faults related to coupled mechanical components (which is RV reducer in this case) using the electrical current signals rather than typically used vibration, acoustic, or ferrography analysis schemes. We presented an approach to detect and diagnose mechanical faults mainly related to the rotate vector (RV) reducer for an industrial robot. The proposed approach utilizes the embedded current signals of the electric motor’s controller to detect the faults, and it provides advantages over typical fault detection and diagnosis methodologies consisting of vibrational, acoustic emission, and ferrography analysis, by (1) eliminating the need to install costly sensors at several locations, and (2) reducing the amount of data involved in the classification process of the faults. Furthermore, this work introduces a feature infusion scheme that focuses on the time-frequency domain analysis of the recorded current signals using DQ0 and DWT with prominent feature selection for ML-based classification. Real-time analysis was performed on an experimental test bench comprising a Hyundai Robot. The classification accuracy achieved for classifying the faults was 96.7%. The results obtained for the proposed approach show that it works well in classifying the faults for RV reducer. In the future, we are working towards the addition of more mechanical components such as strain wave gear and to find a general approach for the fault detection and diagnosis for an industrial robot using AI. 

## Figures and Tables

**Table 1:** Specifications of the electric motors.

Axes No.	Power (kW)	Speed (rpm)	Voltage (V)	Current (A)	Frequency (Hz)
1, 2, 3	5.9	2000	200	25.1	166
4, 5, 6	2	3000	200	11.7	250

**Table 2:** Definition of Wavelet Specific Features.

Feature	Definition
Wavelet Energy	$Energy\;(D_{i})=\overset{N}{\underset{j=1}{\sum }}{\left|D_{ij}\right|}^{2}$ $Energy\;(A_{i})=\overset{N}{\underset{j=1}{\sum }}{\left|A_{ij}\right|}^{2}$
Shannon Wavelet Entropy	$Entropy\;\left(s\right)=s_{i}^{2}log(s_{i}^{2})$

**Table 3:** Definition of Wavelet-based statistical features.

Feature	Definition
Mean	$\mu =\frac{1}{N}\overset{N}{\underset{i=1}{\sum }}n_{i}$
Standard Deviation	$STD=\sqrt{\frac{1}{N}\overset{N}{\underset{i=1}{\sum }}{\left(n_{i}-\mu \right)}^{2}}$
Variance	$VAR=\sqrt{\frac{1}{N-1}\overset{N}{\underset{i=1}{\sum }}{\left(n_{i}-\bar{n}\right)}^{2}}$
Kurtosis	$KRT=\frac{\sum_{i=1}^{N}{\left(n_{i}-\mu \right)}^{4}}{\left(N-1\right)\sigma^{4}}$
Skewness	$SKW=\frac{\sum_{i=1}^{N}{\left(n_{i}-\mu \right)}^{3}}{\left(N-1\right)\sigma^{3}}$

**Table 4:** Detail of the extracted wavelet specific features.

Wavelet Specific Features	D1	D2	D3	D4	D5	D6
$I_{d}$	Energy_Id_D1 Entropy_Id_D1	Energy_Id_D2 Entropy_Id_D2	Energy_Id_D3 Entropy_Id_D3	Energy_Id_D4 Entropy_Id_D4	Energy_Id_D5 Entropy_Id_D5	Energy_Id_D6 Entropy_Id_D6
$I_{q}$	Energy_Iq_D1 Entropy_Iq_D1	Energy_Iq_D2 Entropy_Iq_D2	Energy_Iq_D3 Entropy_Iq_D3	Energy_Iq_D4 Entropy_Iq_D4	Energy_Iq_D5 Entropy_Iq_D5	Energy_Iq_D6 Entropy_Iq_D6

**Table 5:** Detail of the extracted wavelet-based statistical features.

Wavelet-Based Statistical Features	D1	D2	D3	D4	D5	D6
$I_{d}$	Mean_Id_D1 STD_Id_D1 VAR_Id_D1 KRT_Id_D1 SKW_Id_D1	Mean_Id_D2 STD_Id_D2 VAR_Id_D2 KRT_Id_D2 SKW_Id_D2	Mean_Id_D3 STD_Id_D3 VAR_Id_D3 KRT_Id_D3 SKW_Id_D3	Mean_Id_D4 STD_Id_D4 VAR_Id_D4 KRT_Id_D4 SKW_Id_D4	Mean_Id_D5 STD_Id_D5 VAR_Id_D5 KRT_Id_D5 SKW_Id_D5	Mean_Id_D6 STD_Id_D6 VAR_Id_D6 KRT_Id_D6 SKW_Id_D6
$I_{q}$	Mean_Iq_D1 STD_Iq_D1 VAR_Iq_D1 KRT_Iq_D1 SKW_Iq_D1	Mean_Iq_D2 STD_Iq_D2 VAR_Iq_D2 KRT_Iq_D2 SKW_Iq_D2	Mean_Iq_D3 STD_Iq_D3 VAR_Iq_D3 KRT_Iq_D3 SKW_Iq_D3	Mean_Iq_D4 STD_Iq_D4 VAR_Iq_D4 KRT_Iq_D4 SKW_Iq_D4	Mean_Iq_D5 STD_Iq_D5 VAR_Iq_D5 KRT_Iq_D5 SKW_Iq_D5	Mean_Iq_D6 STD_Iq_D6 VAR_Iq_D6 KRT_Iq_D6 SKW_Iq_D6

**Table 6:** Prominent features for Case 2.

No.	Feature
1	SKW_Iq_D6
2	VAR_Id_D6
3	VAR_Iq_D6
4	Mean_Id_D1
5	Mean_Id_D5
6	Mean_Iq_D3
7	STD_Id_D3
8	STD_Iq_D5
9	VAR_Id_D5
10	VAR_Iq_D4
11	Entropy_Id_D6
12	Energy_Iq_D6
13	Entropy_Iq_D6
14	Entropy_Iq_D3
15	Energy_Id_D5
16	Energy_Id_D6
17	Energy_Iq_D4
18	Entropy_Id_D5
19	Energy_Iq_D2
20	Entropy_Id_D1

**Table 7:** Prominent features for Case 4.

No.	Feature
1	Mean_Id_D1
2	Mean_Id_D2
3	STD_Id_D6
4	VAR_Id_D1
5	VAR_Id_D2
6	KRT_Id_D6
7	SKW_Id_D1
8	SKW_Id_D2
9	VAR_Id_D3
10	Mean_Id_D3
11	Energy_Id_D1
12	Energy_Id_D3
13	Energy_Id_D4
14	Energy_Id_D5
15	Energy_Id_D6
16	Energy_Iq_D1
17	Entropy_Id_D3
18	Entropy_Id_D5
19	Entropy_Id_D6
20	Entropy_Iq_D1

**Table 8:** Selected prominent features for Case 5.

No.	Feature
1	SKW_Iq_D6
2	VAR_Id_D6
3	VAR_Id_D6
4	Mean_Id_D1
5	STD_Iq_D5
6	VAR_Iq_D4
7	Entropy_Id_D6
8	Energy_Iq_D6
9	Entropy_Iq_D6
10	Entropy_Iq_D3

**Table 9:** Performance Comparison.

Classifiers	Case 1	Case 2	Case 3	Case 4	Case 5 (Proposed)	Average Performance Score
LDA	66.7%	50%	73.3%	80%	96.7%	73.34
Fine Tree	50%	30%	73.3%	60%	70%	56.66
Naïve Bayes	46.7%	36.7%	36.7%	36.7%	33.3%	38.02
SVM	73.3%	43.3%	70%	86.7%	93.3%	73.32
Average Accuracy Score	59.175	40	63.325	65.85	73.325	
Number of Features	24	60	20	20	10	

**Table 10:** Comparison among proposed and other methods.

Methods	Accuracy
8 Chi-Square Features +Random Forest	93.33%
7 Chi Square Features +SVM	92%
Discrete Wavelet Transform + ANN	93.33%
Statistical Locally Linear Embedding +SVM	94.07%
DWT + Chi square + Correlation Analysis +LDA (Proposed)	96.7%

## References

[1] Lee J., Wu F., Zhao W., Ghaffari M., Liao L., Siegel D. (2014). Prognostics and health management design for rotary machinery systems—Reviews, methodology and applications. Mech. Syst. Signal Process..

[2] Lall P., Hande M., Bhat C., Suhling J., Islam N. (2009). Prognostics and Health Management of Electronics.

[3] Carvalho Bittencourt A. (2014). Modeling and Diagnosis of Friction and Wear in Industrial Robots.

[4] Abichou B., Voisin A., Iung B. (2012). Bottom-up capacities inference for health indicator fusion within multi-level industrial systems. Proceedings of the 2012 IEEE Conference on Prognostics and Health Management.

[5] Sheppard J.W., Kaufman M.A., Wilmering T.J. IEEE Standards for Prognostics and Health Management. Proceedings of the 2008 IEEE AUTOTESTCON.

[6] Yang J., Kim J. (2018). An accident diagnosis algorithm using long short-term memory. Nucl. Eng. Technol..

[7] Zhang L., Lin J., Karim R. (2018). Adaptive kernel density-based anomaly detection for nonlinear systems. Knowl. Based Syst..

[8] Fan J., Yung K.C., Pecht M. (2011). Physics-of-Failure-Based Prognostics and Health Management for High-Power White Light-Emitting Diode Lighting. IEEE Trans. Device Mater. Reliab..

[9] Pecht M., Jie G. (2009). Physics-of-failure-based prognostics for electronic products. Trans. Inst. Meas. Control.

[10] Tsui K.L., Chen N., Zhou Q., Hai Y., Wang W. (2015). Prognostics and Health Management: A Review on Data Driven Approaches. Math. Probl. Eng..

[11] Gao Z., Cecati C., Ding S. (2015). A Survey of Fault Diagnosis and Fault-Tolerant Techniques Part II: Fault Diagnosis with Knowledge-Based and Hybrid/Active Approaches. IEEE Trans. Ind. Electron..

[12] Huang G.-B., Wang D.H., Lan Y. (2011). Extreme learning machines: A survey. Int. J. Mach. Learn. Cybern..

[13] LeCun Y., Bengio Y., Hinton G. (2015). Deep learning. Nature.

[14] Xu Y., Sun Y., Wan J., Liu X., Song Z. (2017). Industrial Big Data for Fault Diagnosis: Taxonomy, Review, and Applications. IEEE Access.

[15] Liao L., Kottig F. (2014). Review of Hybrid Prognostics Approaches for Remaining Useful Life Prediction of Engineered Systems, and an Application to Battery Life Prediction. IEEE Trans. Reliab..

[16] Cerrada M., Sánchez R.-V., Li C., Pacheco F., Cabrera D., Valente de Oliveira J., Vásquez R.E. (2018). A review on data-driven fault severity assessment in rolling bearings. Mech. Syst. Signal Process..

[17] Wang D., Tsui K.-L., Miao Q. (2018). Prognostics and Health Management: A Review of Vibration Based Bearing and Gear Health Indicators. IEEE Access.

[18] Lall P., Lowe R., Goebel K. Prognostics and health monitoring of electronic systems. Proceedings of the 2011 12th Intl. Conf. on Thermal, Mechanical & Multi-Physics Simulation and Experiments in Microelectronics and Microsystems.

[19] Thompson N.C., Greenewald K., Lee K., Manso G.F. (2020). The Computational Limits of Deep Learning. arXiv.

[20] Marsland S. (2014). Machine Learning. Machine Learning.

[21] Silver D., Huang A., Maddison C.J., Guez A., Sifre L., Van den Driessche G., Schrittwieser J., Antonoglou I., Panneershelvam V., Lanctot M. (2016). Mastering the game of Go with deep neural networks and tree search. Nature.

[22] Bojarski M., Del Testa D., Dworakowski D., Firner B., Flepp B., Goyal P., Jackel L.D., Monfort M., Muller U., Zhang J. (2016). End to End Learning for Self-Driving Cars. arXiv.

[23] He K., Zhang X., Ren S., Sun J. (2015). Delving deep into rectifiers: Surpassing human-level performance on imagenet classification. Proceedings of the IEEE International Conference on Computer Vision.

[24] Liu S., Tian Y., Zhang L., Lu B.-L., Kwok J. (2010). Facial Expression Recognition Method Based on Gabor Wavelet Features and Fractional Power Polynomial Kernel PCA. Advances in Neural Networks-ISNN 2010.

[25] Waibel A., Lee K.-F. (1990). Readings in Speech Recognition.

[26] Pazzani M., Billsus D. (1997). Learning and revising user profiles: The identification of interesting web sites. Mach. Learn..

[27] Chan P.K., Stolfo S.J. (1998). Learning with Non-Uniform Class and Cost Distributions: Effects and a Distributed Multi-Classifier Approach. In Workshop Notes KDD-98 Workshop on Distributed Data Mining. http://citeseerx.ist.psu.edu/viewdoc/summary?doi=10.1.1.35.3392.

[28] Guzella T.S., Caminhas W.M. (2009). A review of machine learning approaches to spam filtering. Expert Syst. Appl..

[29] Huang C.-L., Chen M.-C., Wang C.-J. (2007). Credit scoring with a data mining approach based on support vector machines. Expert Syst. Appl..

[30] Randall R.B., Antoni J. (2011). Rolling element bearing diagnostics—A tutorial. Mech. Syst. Signal Process..

[31] Sinha J.K., Elbhbah K. (2013). A future possibility of vibration based condition monitoring of rotating machines. Mech. Syst. Signal Process..

[32] Siegel D., Ly C., Lee J. (2012). Methodology and framework for predicting helicopter rolling element bearing failure. IEEE Trans. Reliab..

[33] Zhen L., Zhengjia H., Yanyang Z., Xuefeng C. (2008). Bearing condition monitoring based on shock pulse method and improved redundant lifting scheme. Math. Comput. Simul..

[34] Cabal-Yepez E., Garcia-Ramirez A.G., Romero-Troncoso R.J., Garcia-Perez A., Osornio-Rios R.A. (2012). Reconfigurable monitoring system for time-frequency analysis on industrial equipment through STFT and DWT. IEEE Trans. Ind. Inform..

[35] Lau E.C., Ngan H.W. (2010). Detection of motor bearing outer raceway defect by wavelet packet transformed motor current signature analysis. IEEE Trans. Instrum. Meas..

[36] Delgado M., Cirrincione G., Garcia A., Ortega J.A., Henao H. A novel condition monitoring scheme for bearing faults based on curvilinear component analysis and hierarchical neural networks. Proceedings of the 2012 XXth International Conference on Electrical Machines.

[37] Jin X., Zhao M., Chow T.W., Pecht M. (2013). Motor bearing fault diagnosis using trace ratio linear discriminant analysis. IEEE Trans. Ind. Electron..

[38] Zhou W., Lu B., Habetler T.G., Harley R.G. (2009). Incipient bearing fault detection via motor stator current noise cancellation using wiener filter. IEEE Trans. Ind. Appl..

[39] Thollon F., Grellet G., Jammal A. (1993). Asynchronous motor cage fault detection through electromagnetic torque measurement. Eur. Trans. Electr. Power.

[40] Elasha F., Greaves M., Mba D., Addali A. (2015). Application of acoustic emission in diagnostic of bearing faults within a helicopter gearbox. Procedia Cirp.

[41] Beguenane R., Benbouzid M.E.H. (1999). Induction motors thermal monitoring by means of rotor resistance identification. IEEE Trans. Energy Convers..

[42] Nejjari H., Benbouzid M.E.H. (2000). Monitoring and diagnosis of induction motors electrical faults using a current Park’s vector pattern learning approach. IEEE Trans. Ind. Appl..

[43] Ondel O., Boutleux E., Blanco E., Clerc G. (2011). Coupling pattern recognition with state estimation using Kalman filter for fault diagnosis. IEEE Trans. Ind. Electron..

[44] Lei Y., Zuo M.J. (2009). Gear crack level identification based on weighted K nearest neighbor classification algorithm. Mech. Syst. Signal Process..

[45] Bechhoefer E., Kingsley M. (2009). A Review of Time Synchronous Average Algorithms. Annu. Conf. Progn. Health Manag. Soc..

[46] Braun S. (2011). The synchronous (time domain) average revisited. Mech. Syst. Signal Process..

[47] He Q., Liu Y., Long Q., Wang J. (2012). Time-Frequency Manifold as a Signature for Machine Health Diagnosis. IEEE Trans. Instrum. Meas..

[48] Portnoff M. (1980). Time-frequency representation of digital signals and systems based on short-time Fourier analysis. IEEE Trans. Acoust. Speech Signal Process..

[49] Tarasiuk T. (2004). Hybrid Wavelet-Fourier Spectrum Analysis. IEEE Trans. Power Deliv..

[50] Anatonio-Daviu J.A., Riera-Guasp M., Floch J.R., Palomares M.P.M. (2006). Validation of a New Method for the Diagnosis of Rotor Bar Failures via Wavelet Transform in Industrial Induction Machines.

[51] Akansu A.N., Medley M.J. (2002). Wavelet, Subband and Block Transforms in Communications and Multimedia.

[52] De Courville M., Xueming L., Duhamel P., Akansu A.N. (1998). Orthogonal transmultiplexers in communication: A review. IEEE Trans. Signal Process..

[53] Rohan A., Kim S.H. (2016). Fault Detection and Diagnosis System for a Three-Phase Inverter Using a DWT-Based Artificial Neural Network. Int. J. Fuzzy Log. Intell. Syst..

[54] Rohan A., Kim S.H. (2019). RLC Fault Detection Based on Image Processing and Artificial Neural Network. Int. J. Fuzzy Log. Intell. Syst..

[55] Rohan A., Rabah M., Kim S.H. (2018). An Integrated Fault Detection and Identification System for Permanent Magnet Synchronous Motor in Electric Vehicles. Int. J. Fuzzy Log. Intell. Syst..

[56] Vinay V., Kumar G.V., Kumar K.P. Application of chi square feature ranking technique and random forest classifier for fault classification of bearing faults. Proceedings of the 22th International Congress on Sound and Vibration.

[57] Konar P., Chattopadhyay P. (2011). Bearing fault detection of induction motor using wavelet and support vector machines (SVMs). Appl. Soft Comput..

[58] Wang X., Zheng Y., Zhao Z., Wang J. (2015). Bearing fault diagnosis based on statistical locally, linear embedding. Sensors.

